# Factors Associated With Tuberculosis as an AIDS-Defining Disease in an Immigration Setting

**DOI:** 10.2188/jea.JE20100072

**Published:** 2011-03-05

**Authors:** Vicente Martín, Patricia García de Olalla, Angels Orcau, Joan A Caylà

**Affiliations:** 1 IBIOMED. Área de Medicina Preventiva y Salud Pública. Universidad de León. CIBER Epidemiología y Salud Pública (CIBERESP), Spain; 2 Servicio de Epidemiología. Agencia de Salud Pública de Barcelona. CIBER Epidemiología y Salud Pública (CIBERESP), Spain

**Keywords:** epidemiology, AIDS, tuberculosis, immigration

## Abstract

**Background:**

Immigration can affect the evolution of TB as an AIDS-defining disease (AIDS–TB).

**Methods:**

The Barcelona AIDS register for 1994–2005 was analyzed, and the global characteristics of AIDS–TB and AIDS–non-TB cases were compared. The Mantel-Haenszel test was used in the trend analysis, and logistic regression was used in the multivariate analysis.

**Results:**

Of the 3600 cases studied, 1130 had both AIDS and TB. A declining trend in AIDS–TB rates was observed in both sexes among both immigrants and native residents. The percentage of AIDS–TB was significantly higher among immigrants (*P* = 0.02). The number of cases among immigrants remained constant over the period of study, but decreased among native residents. The sociodemographic and immunological characteristics associated with TB were male sex, age younger than 36 years, inner city residence, a record of incarceration, greater than 200 CD4+ T-cells/mm^3^, injecting drug use, heterosexual sex, and immigration from Latin America, the Caribbean, or sub-Saharan Africa.

**Conclusions:**

The incidence of TB as an AIDS-defining disease decreased in Barcelona during a recent 10-year period in both native and immigrant populations. However, immigrants remain a high-risk group for AIDS–TB and should be targeted for surveillance and control of both diseases.

## INTRODUCTION

The human immunodeficiency virus (HIV) is the strongest risk factor for the development of tuberculosis (TB) among individuals infected with *Mycobacterium tuberculosis*.^1^ The high prevalence of co-infection by these 2 microorganisms in many geographical areas and in specific population groups has made TB the most common AIDS-diagnostic disease in the world.^2^ For these reasons, the HIV pandemic has modified the epidemiology of TB and necessitated a review of the strategies for TB prevention and control.^3^ One method for evaluating such strategies is to analyze trends in the incidence of TB as an AIDS-defining disease in AIDS registers.^4^^–^^6^

In Spain, TB has been the most common AIDS-defining disease (Centro Nacional de Epidemiología, 2009) since pulmonary tuberculosis was introduced as a diagnostic criterion for AIDS in 1994.^7^ Antiretroviral therapy (ART) and trends in immigration have influenced the epidemiology of these diseases in a number of countries and regions.^8^^–^^10^

The aim of the present study was to examine the factors associated with TB as an AIDS-defining disease in a context where ART is free and universally available and where more than 4 million immigrants have arrived in recent years (Instituto Nacional de Estadística, 2008).

## METHODS

Barcelona, the second largest city in Spain (1 605 602 inhabitants in 2006), is located in the northern part of the east coast of the country. The city AIDS register includes all patients diagnosed with AIDS who were recorded in the Epidemiological Surveillance System, which is an active system for gathering data provided by doctors, hospital discharges, and mortality databases. The register is linked to the registers of TB patients and drug users and thus provides a comprehensive data source.

In this observational, retrospective study of prevalence, we analyzed AIDS cases among city residents older than 13 years who were included in the register between 1994 and 2005. The variables studied were sex, age at AIDS diagnosis, geographical region of origin (Spain, Latin America, and Caribbean; North America and Western Europe; Middle East and North Africa; Sub-Saharan Africa; Rest of Europe and Central Asia; East and South Asia and Pacific), place of residence (inner city or other), period in prison, route of HIV infection (intravenous drug users [IDUs], male non-IDUs who have sex with males, non-IDU heterosexual males, and females and unknown), AIDS-defining disease (AIDS–TB for tuberculosis and AIDS–non-TB for other),^7^ CD4 cell count/mL at diagnosis (≥200, <200, unknown), and date of diagnosis, which was grouped into the periods 1994–1996, 1997–2000, and 2001–2005, which corresponded to the most widely used antiretroviral treatments, ie, pre-HAART, HAART with protease inhibitors, and HAART with non-nucleoside reverse transcriptase inhibitors, respectively. The collected data for AIDS–TB cases were then compared with those for AIDS–non-TB cases. Univariate analysis for categorical and continuous variables was conducted using the chi-square test and the *t* test, respectively. Adjusted odds ratios (ORs) and 95% confidence intervals (CIs) were calculated using logistic regression analysis that included variables associated with AIDS–TB cases with a *P*-value less than 0.2, according to a maximized log-likelihood procedure.

TB rates and 95% CIs for the periods 1994–1998, 1999–2001, 2002–2004, and 2005 were calculated from information provided by the Barcelona City Department of Statistics. The data were obtained from the municipal censuses of, respectively, 1996, 2001, 2004 and 2005 (Ayuntamiento de Barcelona, 2007) and were subdivided by age group, sex, and nationality. Trends were analyzed using the Mantel-Haenszel test for trend.

All data were systematically collected by the AIDS Registry of Barcelona City and were handled in a strictly confidential manner according to the principles of the Declaration of Helsinki, 1964, reviewed and updated by the World Medical Organisation (Edinburgh, 2000). This study also fulfilled the requirements of law 15/1999 on the protection of data, which stipulates that the approval of an ethics committee is not required for this type of analysis.

## RESULTS

A total of 3600 AIDS cases were detected, including 1130 (31.4%) AIDS–TB cases. Localization of TB was exclusively pulmonary in 60.9% of cases (688/1130), exclusively extrapulmonary in 15.0% (169/1130), and mixed in the remaining 24.2% (273/1130). The proportion of cases with smear-positive pulmonary localization was 39.5% (380/961). The time between HIV infection and a diagnosis of AIDS was less than 1 month in 38.4% of native Spaniards and 54.2% of immigrants (*P* < 0.001). Regarding time spent in Spain, 6.2% of immigrants developed AIDS within the 1st year, 46.1% between the 1st and 5th year, 25.9% between the 6th and 10th year, and 21.8% after 10 years. The corresponding distribution was 7.7%, 52.8%, 19.0%, and 20.4% among Latin Americans, and 0%, 52.4%, 21.4%, and 26.2% among sub-Saharans.

The total number of detected cases of AIDS decreased over time among both AIDS–TB and AIDS–non-TB subjects, mainly due to the marked decrease observed among native Spaniards (Table 1).

**Table 1.** Numbers and rates of AIDS cases among residents of Barcelona city by AIDS-defining disease status and place of birth (1994–2005)

Year	Cases						% AIDS–TB			AIDS–TB rate per 100 000 inhabitants		

	AIDS–TB			AIDS–non-TB								
			
	Native	Immigrant	Total	Native	Immigrant	Total	Native	Immigrant	Total	Native	Immigrant	Total
1994	230	15	245	425	32	457	35.1	31.9	34.9	18.0	30.1	18.5
1995	178	11	189	421	27	448	29.7	28.9	29.7	14.0	20.7	14.2
1996	141	6	147	348	14	362	28.8	30.0	28.9	11.1	11.3	11.1
1997	112	9	121	243	16	259	31.5	36.0	31.8	8.8	16.9	9.1
1998	78	7	85	160	7	167	32.8	50.0	33.7	6.1	13.2	6.4
1999	60	8	68	125	9	134	32.4	47.1	33.7	4.7	12.5	5.1
2000	49	11	60	122	9	131	28.7	55.0	31.4	3.9	17.1	4.5
2001	45	7	52	103	19	122	30.4	26.9	29.9	3.5	10.9	3.9
2002	44	10	54	80	12	92	35.5	45.5	37.0	3.6	5.5	3.9
2003	30	15	45	98	21	119	23.4	41.7	27.4	2.5	8.3	3.2
2004	22	13	35	89	16	105	19.8	44.8	25.0	1.8	7.2	2.5
2005	22	7	29	54	20	74	28.9	25.9	28.2	1.8	3.2	2.0

Total	1011	119	1130	2268	202	2470	30.8	37.1	31.4			

Abbreviations: AIDS–TB, tuberculosis as diagnostic disease; AIDS–non-TB, diagnostic disease was not TB; % AIDS–TB, percentage of AIDS–TB with respect to total number of AIDS cases.

**Figure 1.**
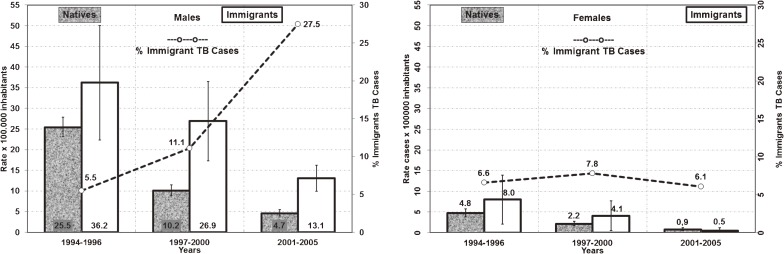
(a) Rates of AIDS–TB cases and immigrant AIDS–TB cases among men, by period and nationality. Error bars represent 95% confidence intervals. Bars: Rate per 100 000 inhabitants. Line: %immigrant TB cases. Native: A person born in or a citizen of Spain. (b) Rates of AIDS–TB cases and immigrant AIDS–TB cases among women, by period and nationality. Error bars represent 95% confidence intervals. Bars: Rate per 100 000 inhabitants. Line: %immigrant TB cases. Native: A person born in or a citizen of Spain.

AIDS–TB cases accounted for approximately 30% of all cases, and no significant change in this rate was observed during the study period. The percentage of AIDS–TB was 30.8% among native Spaniards and 37.1% among immigrants (*P* = 0.02; Table 1). A significant decreasing trend in the percentage of TB was observed among native Spaniards (*P* =0.03), but not among immigrants (Table 1). In 1994, 6.5% of AIDS–TB cases were immigrants, which rose to 37.1% in 2004 (*P* < 0.001). This increase was mainly accounted for by males: 5.5% of AIDS–TB cases in 1994–1996 were male immigrants, which rose to 27.5% in 2001–2005 (Figure 1), while the proportion of female immigrants with AIDS remained between 6.1% and 7.8% (Figure 1).

Among both the native and immigrant groups, AIDS rates also tended to decrease. In 1994, 52.7 AIDS cases per 100 000 inhabitants were registered (18.5 AIDS–TB; 34.2 AIDS–non-TB), which decreased to 7.2 per 100 000 in 2005 (2.0 AIDS–TB; 5.2 AIDS–non-TB). The decrease in AIDS–TB rates was constant throughout the study period: on average, the rate decreased by 20% per year among both natives and immigrants (Table 1).

**Figure 2.**
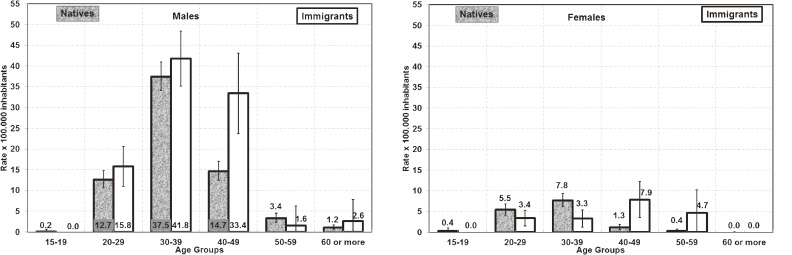
(a) Rates of AIDS–TB cases among men, by age group and nationality. Error bars represent 95% confidence intervals. Native: A person born in or a citizen of Spain. (b) Rates of AIDS–TB cases among women, by age group and nationality. Error bars represent 95% confidence intervals. Native: A person born in or a citizen of Spain.

During the period studied, the average incidence of AIDS–TB declined steadily among males, females, natives, and immigrants, although it remained higher among males and immigrants (Figure 1). The highest AIDS–TB incidence among males was observed in foreign-born men aged 30 to 39 years (Figure 2); among females, the highest incidence was observed in Spanish women aged 30 to 39 years and foreign-born women aged 40 to 49 years (Figure 2).

On multivariate analysis, TB was more common among males, individuals 35 years of age or younger, inner city residents, those with a history of incarceration, those with greater than 200 CD4+ T-cells/mm^3^, IDUs, heterosexuals, and immigrants from Latin America, the Caribbean, and sub-Saharan Africa (Table 2).

**Table 2.** Factors associated with tuberculosis as an AIDS-defining disease (Barcelona 1994–2005)

Variable	No.	TB	%	OR	95% CI	Adjusted OR	95% CI
Sex							
Female	757	204	26.9	1			
Male	2843	926	32.6	1.30	1.1–1.6	1.49	1.2–1.8
Age, yrs							
>35	1743	443	25.4	1			
≤35	1857	687	37.0	1.72	1.5–2.0	1.30	1.1–1.5
Place of residence							
Other districts	2859	824	28.8	1			
Ciutat Vella (inner city)	741	306	41.3	1.74	1.5–2.1	1.73	1.4–2.1
Risk group							
Homosexual	881	160	18.2	1			
IDU	1832	747	40.8	3.10	2.6–3.8	2.58	2.1–3.2
Heterosexual	746	198	26.5	1.63	1.3–2.1	1.96	1.5–2.6
Unknown	141	25	17.7	0.97	0.6–1.6	1.01	0.6–1.7
History of incarceration							
No	2986	780	26.1	1			
Yes	614	350	57.0	3.75	3.1–4.5	2.47	2.0–3.0
CD4 lymphocytes							
<200	1815	475	26.2	1			
≥200	481	250	52.0	3.05	2.5–3.8	3.06	2.5–3.8
Unknown	1304	405	31.1	1.27	1.1–1.5	1.29	1.1–1.5
Period							
1994–1996	1846	582	31.5	1			
1997–2000	1025	334	32.6	1.05	0.9–1.2	1.14	1.0–1.4
2001–2005	729	214	29.4	0.90	0.7–1.1	1.04	0.8–1.3
Region of origin							
Spain	3279	1011	30.8	1			
North America/Western Europe	67	20	29.9	0.94	0.5–1.6	1.06	0.6–1.9
Latin America/Caribbean	142	45	31.7	1.02	0.7–1.5	1.69	1.1–2.5
Middle East/North Africa	48	24	50.0	2.20	1.2–3.9	1.56	0.8–2.9
Sub-Saharan Africa	42	21	50.0	2.20	1.2–4.0	2.39	1.2–4.6
Rest of Europe/Central Asia	11	5	45.5	1.47	0.2–8.8	0.94	0.3–3.4
South Asia/East Asia/Pacific	11	4	36.4	1.10	0.2–6.0	1.17	0.3–4.4

Abbreviations: CI, confidence interval; IDU, intravenous drug user; OR, odds ratio; TB, tuberculosis.

## DISCUSSION

The 1994 adoption of pulmonary TB as an AIDS-defining disease among individuals infected with HIV^7^ resulted in the highest number of detected cases in the European Union (93.7 per million inhabitants), Spain (183.5 cases per million), and Barcelona (464.3 cases per million).^14^ Since that year, there have been consistent decreases in both the number of cases and the incidence of AIDS. As compared with 1994 values, incidence in 2005 was 75% lower (<20 cases per million) in the European Union, 80% lower (36 cases per million) in Spain, and approximately 90% lower (68.7 cases per million) in Barcelona. The marked declines in both AIDS–TB and AIDS–non-TB cases have been attributed to improvement in the immune status of HIV-infected individuals owing to highly active antiretroviral therapies (HAART) and the effectiveness of programs for prevention and control of HIV infection and TB.^11^^,^^12^ The reduction in AIDS–TB rates observed in our study is likely attributable to the same causes. In Spain, ART has always been widely accessible and free, and, since 1997, approximately 70% of HIV-infected individuals have been receiving HAART.^13^^–^^15^ HIV prevention and control activities in the city of Barcelona have been effective, especially among IDUs.^16^ The tuberculosis control program is also effective and may partly explain the decline in AIDS–TB cases that occurred during the pre-HAART period.^17^

Regarding TB as an AIDS-defining disease, Barcelona has historically had high incidences of AIDS and TB, with many cases of comorbidity, which explains why more than 30% of AIDS cases had TB as their AIDS-defining disease. This percentage is higher than those observed in Central and Western Europe (20% and 25%, respectively),^18^ the United States (5%),^19^ France (10%),^20^ Brazil (24%),^21^ London (19%),^4^ and New York (5.3%),^22^ and lower than incidences observed in Eastern Europe (50%)^18^ and Portugal (51%),^18^ where the incidence of TB is higher and lower, respectively.

As in previous studies, the factors predicting the presence of TB as an AIDS-defining disease are consistent with the high observed prevalences of co-infection by HIV and *M. tuberculosis* and are associated with the same population groups: males, young people, IDUs, promiscuous heterosexuals, individuals with a history of incarceration, and immigrants from countries with a high prevalence of latent tuberculosis infection (LTBI).^23^^,^^24^ It should be noted that, in Barcelona, the inner city is the district with the lowest socioeconomic level and the city’s highest incidences of TB and AIDS.^25^ The present study shows that a level of greater than 200 CD4+ T-cells/mm^3^ is associated with the presence of TB, which could be due to the high prevalence of LTBI.^25^^,^^26^ The absence in the city of outbreaks, which are associated with the presence of individuals with severe immunosuppression, indicates that endogenous reactivation and treatment of LTBI may be important in contexts where the prevalence of co-infection is high.^27^^,^^28^

In the present study, immigrants from Latin America, the Caribbean, and sub-Saharan Africa were more likely to develop TB as an AIDS-defining disease—as was observed in previous studies in countries with high levels of immigration from those geographical areas—probably because of the high prevalence of TB-AIDS co-infection in their countries of origin.^8^^,^^29^^,^^30^ However, the endemic nature of TB-AIDS co-infection in countries of origin may not be sufficient to explain this finding. In the case of immigrants with latent tuberculosis infection (LTI) alone, the social group into which immigrants integrate in the receiving country could determine their risk of HIV infection and, therefore, the occurrence of AIDS–TB cases.^31^ On arrival in Spain, some immigrants become IDUs or sell sex for money, thereby increasing the risk of HIV infection.^31^

AIDS–TB rates observed among immigrants were higher than among native Spaniards, but may be overestimated because of a higher real denominator due to the existence of illegal immigration. Nonetheless, other cohort studies have also found higher rates among immigrants.^30^ It should also be noted that access to methods of early detection of HIV infection may have been impaired among immigrants, as was observed in the present study and in other Spanish studies, perhaps due to the illegal residence status of some immigrants and the fact that HIV transmission occurred mainly among heterosexuals, a group perceived as less at risk.^14^^,^^15^^,^^30^^,^^34^ The proportion of AIDS–TB cases increased among immigrants because the number of cases among that population remained constant over time, which was not the case among native Spaniards. A similar tendency was observed in the Spanish HIV infection registry^32^ and the Lazio TB registry in Italy.^33^

Immigration in Spain has increased considerably in recent years: immigrants formed 2.3% of the population in 2000 and 8.5% in 2005 [Instituto Nacional de Estadística, 2008]. In Barcelona, the number of immigrants rose from 29 534 in the 1996 census to 260 058 in 2005 (16% of the population; Ayuntamiento de Barcelona, 2006). The vast majority come from countries with higher TB rates and lower HIV infection rates than Spain (mainly countries in Latin America, Eastern Europe, and North Africa). This could explain the low impact of immigration on the rate of AIDS–TB in Spain, in contrast to trends observed elsewhere, where the decline in AIDS–TB rates has been checked by the arrival of immigrants from areas of high co-infection, particularly sub-Saharan Africa.^12^^,^^29^ These findings must be interpreted with caution, however, as an immigrant population is not normally representative of its country of origin and does not reflect its epidemiological patterns. Nonetheless, it is not difficult to imagine an exchange of infections that might occur between one group with a high prevalence of LTBI and low HIV infection (immigrants) and another with lower LTBI prevalence but a higher rate of HIV infection (natives).^31^ This hypothesis is supported by the fact that immigrants who had been in Spain longer had developed HIV infection in that country. It thus seems likely that approximately half the comorbid immigrants arrive in Spain with co-infection, and at least one third become co-infected while living in Spain.^31^

## CONCLUSIONS

The incidence of TB as an AIDS-defining disease decreased in Barcelona during 1994–2005 in both the native and immigrant populations. To ensure that this trend continues in the future, it is essential to intensify HIV infection and TB control programs specifically directed at those immigrant groups most at risk of HIV infection (ie, drug users, sex workers, the promiscuous, etc.).
